# How Does a Junior Faculty Development Program Affect Burnout? A Mixed Methods Assessment

**DOI:** 10.1177/23821205231223294

**Published:** 2024-02-04

**Authors:** Timothy D Riley, Jessica A Parascando, Erika VanDyke, Heather L Stuckey, Huamei Dong, Omrana Pasha-Razzak, Lawrence E Kass, Jennifer McCall-Hosenfeld, Sarah K Bronson

**Affiliations:** 1 Department of Family and Community Medicine, 12310 Penn State College of Medicine, Hershey, PA, USA; 2 Department of Medicine, 12310 Penn State College of Medicine, Hershey, PA, USA; 3 Division of General Internal Medicine, Department of Medicine, Humanities and Public Health Sciences, 12310 Penn State College of Medicine, Hershey, PA, USA; 4 Department of Public Health Sciences, 12310 Penn State College of Medicine, Hershey, PA, USA; 5 Department of Emergency Medicine, 12310 Penn State College of Medicine, Hershey, PA, USA; 6 Department of Public Health Sciences, 12310 Penn State College of Medicine, Hershey, PA, USA; 7 Department of Cellular and Molecular Physiology, 12310 Penn State College of Medicine, Hershey, PA, USA

**Keywords:** Burnout, wellness, faculty development, qualitative research

## Abstract

**OBJECTIVES:**

Burnout is common among junior faculty. Professional development has been proposed as a method to improve engagement and reduce burnout among academic physicians. The Penn State College of Medicine Junior Faculty Development Program (JFDP) is a well-established, interdisciplinary program. However, an increase in burnout was noted among participants during the program. The authors sought to quantify the change in burnout seen among JFDP participants across 3 cohorts, and to explore sources of well-being and burnout among participants.

**METHODS:**

Through a sequential explanatory mixed methods approach, participants in the 2018/19, 2019/20, and 2020/21 cohorts took a survey assessing burnout (Copenhagen Burnout Inventory), quality of life (QoL), job satisfaction, and work–home conflict at the start and end of the course. Descriptive statistics were generated as well as Pearson χ^2^ test/Fisher exact test for categorical variables and Wilcoxon rank sum tests for continuous variables for group comparisons. To better understand the outcome, past participants were invited to interviews regarding their experience of burnout during the course. Inductive thematic analysis (kappa = 0.86) was used to derive themes.

**RESULTS:**

Start- and end-of-course surveys were completed by 84 and 75 participants, respectively (response rates: 95.5% and 85.2%). Burnout associated with patient/learner/client/colleague increased (*P* = .005) and QoL decreased (*P* = .02) at the end compared with the start. Nonsignificant trends toward worsening in other burnout categories, work–home conflict, and job satisfaction were also observed. Nineteen interviews yielded themes related to risks and protective factors for burnout including competing demands, benefits of networking, professional growth, and challenges related to diverse faculty roles.

**CONCLUSION:**

Junior Faculty Development Program participants demonstrated worsening of burnout and QoL during the program while benefiting from opportunities including skill building and networking. The impact of Junior Faculty Development Programs on the well-being of participants should be considered as an element of their design, evaluation, and refinement over time.

## Introduction

Burnout is common among faculty in academic health centers (AHCs)^1–3^ and is associated with significant personal and professional consequences.^3,4^ Early career faculty are at higher risk for burnout.^5,6^ Faculty development has been proposed as a method to address burnout among academic faculty,^7–9^ with some programs showing benefits in burnout-related factors.^10–12^ However, the impact of professional development programs on burnout among junior faculty at AHCs is not yet well-defined.

The Penn State College of Medicine (PSCOM) Junior Faculty Development Program (JFDP) is a well-established, interdisciplinary program.^13,14^ We routinely measured burnout among JFDP participants at the start and near the end of the program to foster self-reflection and group dialogue on the topic. We found an apparent increase in burnout later in the program and sought to understand these findings.

We aimed to quantify the change in burnout seen among JFDP participants in 3 consecutive cohorts, and to explore sources of well-being and burnout among participants through a sequential explanatory mixed-methods approach.

## Methods

This study received IRB approval from the Penn State Human Research Protection Program (HRPP) on July 16, 2020 (Study #15576). Verbal informed consent was approved by the Penn State HRPP and was provided by each participant prior to participation.

### Study Design

This mixed-methods study utilized a sequential explanatory design. The interview portion of the study was designed and executed after survey completion.

### Program

A curriculum was designed in 2003 for junior faculty to encourage inquiry, collaboration, and improvements in participants’ academic careers.^14^ Participants are selected through a competitive application process, with approximately 90% of applicants accepted. Applicants must have a letter from their chair indicating support for their intended project and a guarantee of 0.1 full-time equivalent (FTE) dedicated time. Though the specific content has evolved over the years, the essential elements have remained constant. Participants meet for 2 to 3 hours every Friday morning at 7 am over the course of 9 months (September to May) covering topics important to academicians including research/scholarship, teaching, professional identity formation, and professional development (Table 1). Beginning in 2018 to 2019, well-being was addressed in the curriculum during 2 workshops led by authors TR and JMH, one in the first half of the academic year (fall) and another in the second half (spring). As part of the well-being sessions, burnout levels were routinely assessed at each of these and used for reflection and dialogue in class. The program also emphasizes a longitudinal research project and an interdisciplinary mentorship structure. Both clinical and basic science faculty participate, and mentors must be from a different department than their mentee. The mentors are selected by program leadership from a pool of experienced faculty after discussion with the participant to identify their particular needs from a mentor. To complete the program, participants must contact and schedule meetings with their assigned mentor, attend at least 80% of the scheduled program elements, participate in 3 required sessions (with make-up sessions offered if needed), and submit key deliverables (Table 2).

**Table 1.** Junior Faculty Development Program Curricular Elements.

Research and Scholarship	
Identifying Opportunities for Scholarship	Peer Review of Proposals
Concept Models and Concept Papers	Effective Posters
Brainstorming and Refining Scholarly Questions	Turning QI into Scholarship
Step-Back Analysis	Publishing a Manuscript
Navigating Project Resources and Oversight	Project Poster Presentation
Proposal Writing	Project Presentations – Practice
Proposal Review & Mock Review	Project Presentations – Formal (End of Year)
Educator/Teaching	
Perspectives on Teaching & Learning	Teaching-Putting it All Together
Adult Learning Theories	Focused Teaching Project
Creating a Curriculum	
Professional Identity Formation	
Core Values/Setting Goals	Conversations with Leadership
Working in Teams	Diverse Groups: Unconscious Bias & Micro-aggression
Resilience and Engagement	
Professional Development	
Time Management	Promotion and Tenure Discussion and Panel
Systems Thinking	Academic Compensation in Clinical and Basic Science Departments
Manuscript Review and Ethics	Negotiation Panel & Annual Review Role Play
Effective Professional Documents: CV, Biosketch & Educator Portfolio	

**Table 2.** Junior Faculty Development Program Participant Expectations.

**Program Expectations**
Write a brief project proposal
Obtains letter of support from chair
Obtain training in the required learning management system
**Mentor–Mentee Relationship Expectations**
Mentees contact mentors to set schedule and agree on meeting frequency and content
**Attendance Expectations**
Attend at least 80% of sessions
* The following sessions are required (make-up sessions available):*
Step Back Analysis
Peer Review Session
Focused Teaching Session
Required Deliverables
Project progress report (October)
Draft of project support proposal (December)
Revised project support proposal (February)
Project poster presentation (February)
Final project support proposal (April/May, optional)
End of year oral presentation (April/May)
**Additional Activities**
Program kickoff (August/September)
Program graduation (May/June)
Year 2 meetings (quarterly, optional)

### Participants

We recruited participants based on the following inclusion criteria: (1) graduate members of the JFDP program at PSCOM from the 2018/19, 2019/20, and 2020/21 cohorts (n = 88), (2) adults aged 18 years or older, and (3) fluent in written and spoken English. JFPD participants from pre-2018 cohorts were excluded**.** As a result of COVID-19 restrictions, the second cohort shifted to a virtual format after March 2020, and the third cohort was fully virtual.

### Quantitative Evaluation

Participants from Cohorts 1 (2018/19), 2 (2019/20), and 3 **(**2020/21) completed quantitative assessments of burnout and well-being during time set aside for the surveys in the fall and spring workshops focused on well-being. Cohort 3 was added to the quantitative analysis due to the availability of routinely collected data after qualitative interviews had been completed. The following were assessed in the survey using validated questions/questionnaires: (1) burnout using the Copenhagen Burnout Inventory,^15^ which categorizes burnout as related to personal, work and client-based factors with scores >49 signifying high burnout^16^; (2) quality of life (QoL) using a scale from 0 “as bad as it can be” to 10 “as good as it can be,” presented as a mean score^6^; (3) job satisfaction using a scale from 0 “very little” to 6 “very much,” with scores >3 signifying high job satisfaction^17^; and (4) work–home conflict (WHC) using a scale from 1 “strongly disagree” to 5 “strongly agree,” with scores <4 signifying high WHC.^3^ For the purposes of this study, considering the interdisciplinary nature of the participants including clinicians from multiple specialties and basic scientists, the “clients” category was expanded to include patients, learners, clients, and/or colleagues. Details regarding quantitative assessment and scoring thresholds of burnout, QoL, job satisfaction, and WHC are listed in Table 3. Open-ended questions were asked related to drivers of dissatisfaction and burnout, key elements to sustaining well-being, and other comments. Demographics were also asked. Participation was voluntary.

**Table 3.** Quantitative Assessment of Burnout, Quality of Life, Job Satisfaction, and Work–Home Conflict.

Survey element	Number of questions	Scale	Thresholds
Copenhagen Burnout Inventory (CBI)^15^^a^	19	5-point Likert scale responses (“never/almost never or to a very low degree” to “always or to a very high degree”)	Scores greater than 49 were considered high burnout for each category.^16^
Quality of life^6^	1	Rated on a scale from 0 (“as bad as it can be”) to 10 (“as good as it can be”).	Mean score
Job satisfaction^17^	1	Rated on a scale from 0 (“very little”) to 6 (“very much”)	High job satisfaction being defined as a score of >3.
Work–Home Conflict^3^	1	The degree to which their work schedule left them enough time for personal/family life on a scale from 1 (“strongly disagree”) to 5 (“strongly agree”)	High work–home conflict being defined as a score of <4.

^a^Categorizes burnout related to personal, work, and clients. For the purposes of this study, considering the interdisciplinary nature of the participants including clinicians from multiple disciplines and basic scientists, the “clients” category was expanded to include patients, learners, clients, and/or colleagues.

### Qualitative Evaluation

The authors followed the Standards for Reporting Qualitative Research framework.

**Figure 1.**
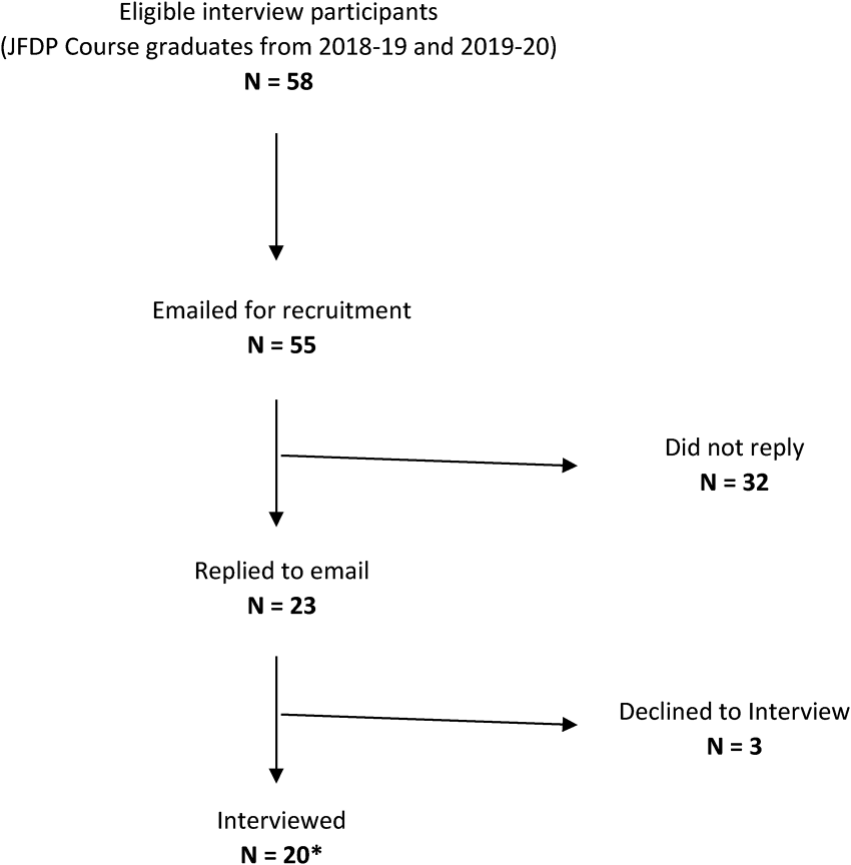
Consolidated Criteria for Reporting Qualitative Studies (COREQ) Flow Diagram.

Interview participants from Cohorts 1 and 2 were recruited via email. A convenience sample of interview participants were selected on a first-come, first-serve basis until saturation was reached (Figure 1). As saturation was reached during interviews with Cohorts 1 and 2, it was not necessary to pursue additional interviews with Cohort 3.

**Figure 2.**
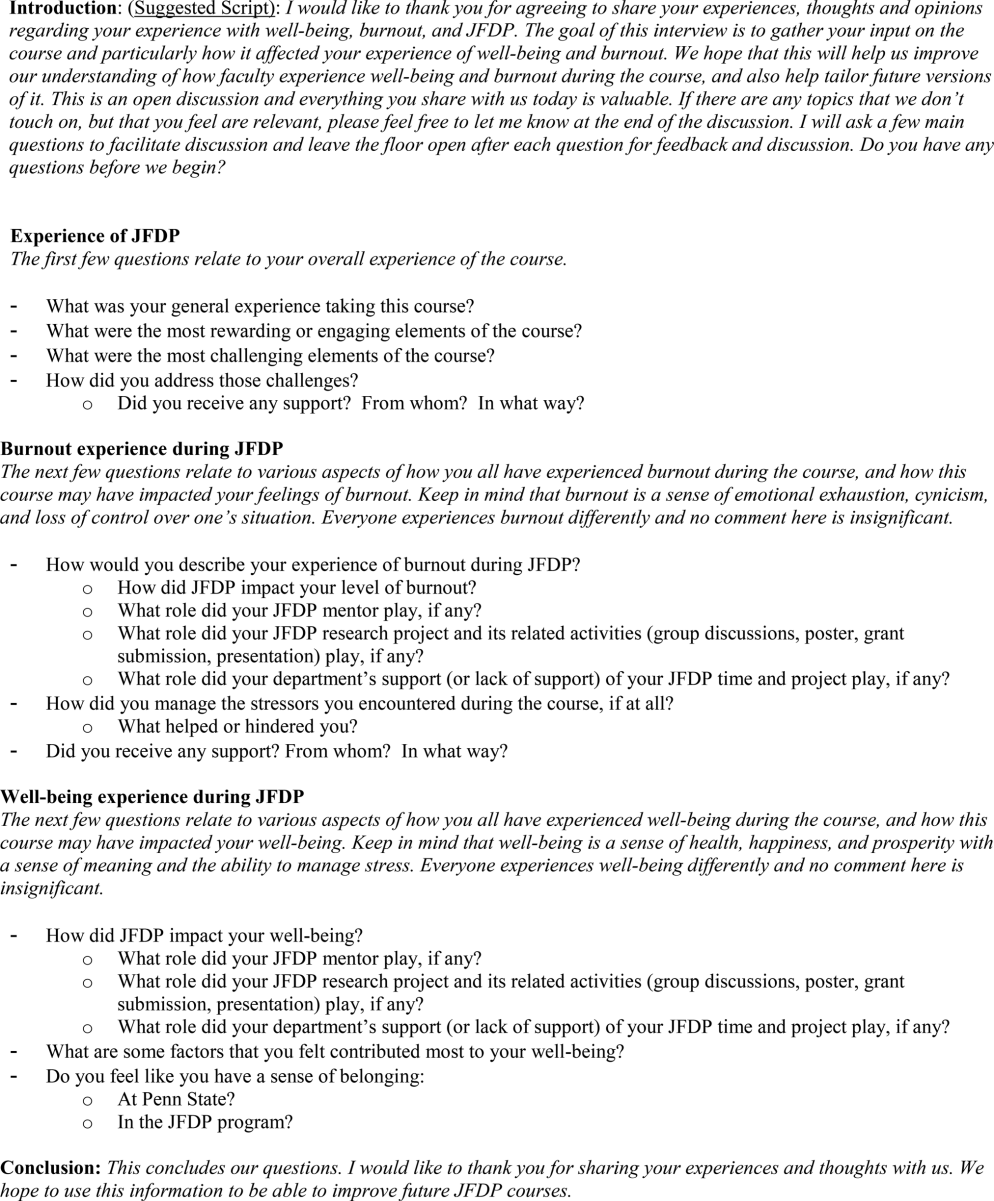
Semistructured Interview Guide.

Study author JP conducted 15- to 20-min, one-on-one, audio-recorded telephone interviews using a semistructured interview guide (Figure 2) between Nov sixth and December 23, 2020. All interviews were done with participants who had completed the program approximately 6 to 18 months prior. We felt those who had completed the program would have a perspective on its full scope and its effects on their career trajectory. Recent graduates were recruited to optimize recall of program impact on well-being. Audio files of the interviews were professionally transcribed with all identifiers removed.

### Data Analysis

#### Quantitative

Anonymity prevented pre-post analysis of each individual's responses. All data was grouped into “fall” (near the start of the program) and “spring” (near the end of the program). Quantitative analysis of outcomes was performed using the R statistical program (version 4.0.2) to generate reproducible statistical analyses and tables. Basic descriptive statistics were generated, as well as Pearson χ^2^ test/Fisher exact test for categorical variables and Wilcoxon rank sum tests for continuous variables.

#### Qualitative

Thematic analysis describes the systematic process for coding data that was used to answer the research question.^18^ Study team members (TR, HS, JP, and EV) reviewed transcripts line-by-line to develop relevant codes reflecting participant experiences within JFDP. Using NVivo Version 12, 2 coders (JP and EV) coded 20% of the transcripts (n = 4) using the codebook. Reliability and agreement of individual coding was compared between the coders. After adjustments were made to the codebook, coders reached an acceptable kappa (k = 0.86). Using the finalized codebook, coder JP coded the remaining transcripts. Coded transcripts were shared with study team members (TR, HS, JP, and EV) who discussed initial emerging themes. Coded data were reviewed and themes finalized in a second meeting with the larger research group.

## Results

### Quantitative

Eighty-four and 75 participants completed the fall and spring surveys, respectively (response rates: 95.5% and 85.2%). For both survey periods, participants were mostly female, white non-Hispanic, early in their careers (<5 years working), in their mid to upper 30s, and had total FTE ≥ 0.90 and clinical FTE < 0.90 (Table 4).

**Table 4.** Junior Faculty Development Program Participant Demographics Arranged by Assessment Period (Fall, n = 84; Spring, n = 75) Between 2018 and 2021.

Variable	Fall surveys 2018-2020	Spring surveys 2019-2021
	n = 84	n = 75
Response Rate	95.5%	85.2%
Sex, n (%)		
Male	24 (28.6)	21 (28.0)
Female	46 (54.8)	34 (45.3)
Prefer not to answer	0 (0)	2 (2.7)
No answer	14 (16.6)	18 (24)
Race/Ethnicity, n (%)		
Asian	14 (16.6)	11 (14.7)
Black or African American	2 (2.4)	1 (1.3)
Hispanic	4 (4.8)	3 (4.0)
Middle Eastern	1 (1.2)	1 (1.3)
White non-Hispanic	39 (46.4)	31 (41.3)
Other	5 (5.9)	0 (0)
Prefer not to answer	4 (4.8)	5 (6.7)
No answer	15 (17.9)	23 (30.7)
FTE, n (%)		
≥90%	33 (39.3)	28 (37.4)
<90%	7 (8.3)	7 (9.3)
Prefer not to share	1 (1.2)	0 (0)
Not applicable	1 (1.2)	0 (0)
No answer	42 (50)	40 (53.3)
Clinical FTE, N (%)		
>=90%	7 (8.3)	2 (2.7)
<89%	32 (38.1)	31 (41.3)
No answer	45 (53.6)	42 (56.0)
Years working, M (SD)	2.5 (2.4)	3.1 (2.6)
No answer, n (%)	18 (21.4)	23 (30.7)
Age in years, M (SD)	36.6 (4.9)	37.2 (4.9)
No answer, n (%)	18 (21.4)	26 (34.7)

Results (Table 5) showed significantly increased patient/learner/client/colleague burnout (*P* = .005) and significantly decreased QoL (*P* = .02) in the spring compared with the fall. Nonsignificant trends toward worsening in other burnout categories, WHC, and job satisfaction were also observed.

**Table 5.** Summary of Outcome Variables for Fall (n = 84) Versus Spring (75) for Junior Faculty Development Program Participants Between 2018 and 2021.

	Fall	Spring	*P* value^a^
	n = 84	n = 75	
Burnout, n (%)			
Work >49	12 (14.3)	17 (22.7)	.25
Home >49	19 (22.6)	23 (30.7)	.33
Patient/learner/client/colleague >49	8 (9.52)	21 (28.0)	.005
Work–home conflict < 3 n (%)	20 (23.8)	24 (32.0)	.33
Quality of life, mean (SD)	6.82 (1.88)	6.13 (1.92)	.02
Job Satisfaction >3, n (%)	64 (76.2)	49 (65.3)	.18

^a^ *P* value for between group comparison using Pearson χ^2^ tests/Fisher exact tests for categorical variables and Wilcoxon rank sum tests for continuous variables.

### Qualitative

Saturation of data was reached after 20 interviews. Nine participants were interviewed from Cohort 1, and 11 from Cohort 2. One recording was lost due to a technological error, therefore, only 19 interviews were used in the analysis. Overall, participants found JFDP to be helpful in their growth as faculty, and would recommend the program to other faculty. There were 4 themes (Tables 6 and 7), describing positive rewards of the program and suggesting possibilities for improvements and barriers that may have led to a greater sense of burnout.

**Table 6.** Codes Used to Support Theme Development for Junior Faculty Development Program Interviewees (n = 19).

Code	Definition of code	# of Part^a^	# of Refs^b^
**Theme 1: Participation in the program led to personal growth including improvements in knowledge, scholarly skills, and motivation to continue research.**			
Personal growth and development	Individuals’ cited growth through the JFDP program that positively impacted wellbeing, development of new skills, and accomplishment of projects and professional goals.	15	33
Individual drive/motivation	Discussion of motivation to continue to work on projects outside of the program, and personal drive that led an individual to reach program and professional goals.	5	6
*Knowledge gained*			
Institutional	Learning about Penn State practices, services, and resources, (eg, IRB, promotion, academia, health systems).	9	14
Research	Learning about the different types of research processes, different stages of scholarly activity, available resources and transferable skills.	11	20
Other	Learning about other tools and skills such as education and teaching strategies.	9	14
**Theme 2: Participants appreciated networking opportunities and support at multiple levels, and felt that the program would be beneficial to others.**			
Networking and/or Interaction with peers	References to connections and collaborations made with their JFDP cohort, mentors, and people from other departments.	18	63
Positive aspects of the curriculum	Found curriculum topics, components and materials useful, informative and enjoyable.	17	50
Would recommend to colleagues	Expresses they would recommend to early career faculty.	19	24
*Support*			
Mentor	Positive interactions with assigned and informal mentors.	14	38
Department	Notations of departments that supported the individual in completing JFDP program requirements through protected time and interaction with department JFDP alumni.	17	48
JFDP environment	Describes a “safe place” for learning, positive and encouraging colleagues, Comradery and personalized feedback from other participants.	16	39
Other Support	Support from outside of work and the program.	1	2
**Theme 3: Competing program requirements, clinical work, and family responsibilities left participants feeling overwhelmed.**			
JFDP Project Demands	Discussion of stress related to project components such as the IRB process, completing necessary forms, project demands such as grant writing and poster presentation, meeting deadlines, honing in on scope of project, and time to actually complete project.	15	43
Burnout/stress	Expression of emotional toll of project, or feeling overwhelmed, tired or frustrated from having to balance clinical and academic duties, and family/home life.	11	28
*Competing Demands*			
Work and/or professional	Competing work demands such as clinic time, and teaching responsibilities contributed to burnout, or negatively impacted JFDP experience.	16	35
Family	Home and family responsibilities such as childcare and managing the home contributed to burnout, or negatively impacted JFDP experience.	2	5
**Theme 4: Program characteristics, such as its breadth, timing, and reliance on supportive mentors and departments contributed to burnout and frustration.**			
Mentorship inconsistent/lacking	Negative interactions with assigned mentor.	9	25
Insufficient personal time management	Individual lack of time management skills and balance and organization of work and home responsibilities.	5	8
Unsupportive departments	Notations of departments that were not supportive during JFDP experience, including having to use administrative or time outside of work to complete program requirements.	6	9
COVID related factors	Any COVID-19 specific factors that contributed to burnout or any mentions of COVID-19 factors that negatively impacted the JFDP experience, such as cut in administrative time and delays in projects.	7	21
Lack of prior research knowledge	Comments from those new to research who felt unprepared for the project requirements due to lack of skillset.	4	10
Overall negative experience	Negative remarks about the program, unfulfilled expectations, and adjustment to their new role in the institution.	4	11
Negative aspects of the curriculum	Desire for more relevant role related topics such as inclusion of education, curriculum development, wellness and faculty development related topics, and more time to connect with participants.	12	42
Program Timing	Lack of enthusiasm and challenges associated with the early start time of the program.	5	7

^a^ # of participants = the number of participants with quotes that could be coded here.

^b^ # of references = the number of times this code was referenced; that is, one participant could have mentioned more than one topic that was coded here. None of the data were double-coded, meaning a quote was only coded once, to one code only.

**Table 7.** Qualitative Themes and Representative Quotes From Junior Faculty Development Program Interviewees (n = 19).

Themes	Representative quotes
Theme 1: Participation in the program led to personal growth including improvements in knowledge, scholarly skills, and motivation to continue research.	*[JFDP] offered me opportunities to learn how to do more aspects of scholarly activity that I previously did not have the skillset to do.*
	*It offered me the chance to really plan out all the potential stages of the project, [and] to have a sounding board to go to think through the different barriers that are commonly encountered and come up with strategies to try to solve them. To practice putting together a presentation, and grant writing, and realize [the] resources that are available here. And to think a little bit more about what it means to be an academic physician, what the academic part of it really means.*
	*It offered me a way to learn about different types of education and teaching.*
	*The program offered some context of the health system, which helped as far as thinking about my role within the health system, resources and things like that.*
Theme 2: Participants appreciated networking opportunities and support at multiple levels, and felt that the program would be beneficial to others.	*During the time of JFDP, I think the department was very supportive. That was a huge plus and without that support, I wouldn't have gotten as far as I did.*
	*Having some other people who are positive about scholarly activity around me was helpful.*
	*The support of a mentor who's been through the process before was invaluable and the opportunity to meet colleagues, because you never know who may be a good person to do a shared project with in the future or learn a new idea that you may be able to adopt to your clinic and I think for those reasons it's really invaluable.*
Theme 3: Competing program requirements, clinical work, and family responsibilities left participants feeling overwhelmed.	*There was a time when I felt a bit overwhelmed with my project and I got totally sick of thinking about it. I'm like, “Oh my gosh, this is just so tiresome and terrible and I just want to give up.” And I just didn't even want to think about my project, I was just sick and tired of thinking about it, because I thought, “This is too hard, it's too hard.”*
	*I felt like there was a lot of pressure put from the aspect of doing research and writing a paper and submitting grants that was all centered on the project, so I did feel some pressure throughout that for me. It's good knowledge to have going forward, but I found it a little bit stressful, as a participant, how much pressure was being put on this aspect of it, when that's not 100% what I went into the program looking for.*
	*I'm still having some difficulty with getting the project even off the ground. Like getting that form that I needed to submit my IRB for the project. Submitting the IRB was a definite stressor.*
	*I relocated, I had a baby a couple months before, my husband was trying to get a new job. So there were a lot of extra things going on and trying to get my feet wet from a clinical standpoint, plus the balances JFDP. So I think my experience may be unique because of my other factors going on, but it was a little bit challenging to balance it along with everything else that was brand new.*
Theme 4: Program characteristics, such as its breadth, timing, and reliance on supportive mentors and departments contributed to burnout and frustration.	*There was no time for any personal connection. So it was just like, you go on, it's early, not everybody's an early person. So people are just trying to get their coffee and try to settle down.*
	*I know different departments and divisions handle this differently, because my wife went through the program and she did have a 10% clinical buydown and a 5% clinical buydown. So I was a little bit surprised when I found out, even in the same department but a different division, that no, in fact I was not having that happen.*
	*I found the most challenging was I don't have prior experience in doing research. Some of the prompts were a little bit unclear as far as what we were supposed to do, which caused a lot of us to scramble at the last minute, [or] the night before. Trying to do some of this writing as well, I think, was a challenge.*
	*I ended up quitting my project because I felt I wasn't connecting with my mentor.*

Theme 1: Participation in the program led to personal growth including improvements in knowledge, scholarly skills, and motivation to continue research.

Participants gained skills in research and knowledge of resources available at the institution. Participants also gained confidence in these skills allowing for personal growth and development, and motivation to pursue their interests after completing the program.

“*I remember leaving those morning JFDP sessions just feeling the sense of enthusiasm and excitement for where I work and my goals for myself. … I have these exciting goals and I feel like a meaningful part of this institution and that's my decision.”*

Theme 2: Participants appreciated networking opportunities and support at multiple levels, and felt the program would be beneficial to others.

The participants described their appreciation of support inside and outside the program. Overall participants had a positive experience in the program and would recommend it to their colleagues.

“*My mentor and I [are] still [in] contact regularly for things that have nothing to do with JFDP, and just learning different faces around the hospital. So that would be my top ‘why’ to participate.”*

Theme 3: Competing program requirements, clinical work, and family responsibilities left participants feeling overwhelmed.

Difficulty of managing work–life responsibilities often left participants spending time working outside of dedicated JFDP time to complete responsibilities. This increased feelings of stress and burnout. Some found it difficult to complete program requirements while managing other responsibilities and more intense projects.

“*I think the balance of work, academics and family responsibilities created a sense of feeling overwhelmed and helpless at times.”*

Theme 4: Program characteristics, such as its breadth, timing, and reliance on supportive mentors and departments contributed to burnout and frustration.

Participants expressed frustration and discordance regarding their expectations of the program. While some participants noted their lack of skills in research basics or poor time management, others blamed COVID-19-related factors, institutional barriers, or unsupportive work environments.

“*And just with the lack of skillset going into that made it even harder. I think for some people who were already PhDs in research, this was a breeze for them to do a grant application.”*

## Discussion

Participants in this program demonstrated significant worsening of patient/client/learner/colleague burnout and QoL during the program. Trends toward worsening work and personal burnout, job satisfaction, and WHC were also observed. These findings contrast with prior reports suggesting improvement in well-being related factors associated with professional development,^10–12^ and raise questions about the possibility of using it to address burnout.^7^

Qualitative findings suggest the challenges of increased workload and incorporating new skills were the main drivers of increased stress and burnout (Theme 3). Interviewees articulated that the program increased their workload and created difficulty in managing assignments with personal and professional responsibilities. The onset of COVID-19 during the study period likely impacted demands related to teaching, clinical care, and research. Although all chairs were asked to reaffirm their commitment to each JFDP participant's protected time, several participants reported to the program leadership that they were unable to attend JFDP sessions or fully participate due to increased or unanticipated clinical demands. The pandemic also resulted in worsening burnout for clinicians across the United States.^19,20^ We were unable to control for this national trend in our study. Interviews took place in November to December 2020, and participants’ experiences related to COVID may have influenced their responses in ways that were not accounted for in the qualitative analysis. Given participants’ reports of difficulties balancing program assignments with personal and professional responsibilities, increasing demands related to the pandemic may have exacerbated existing challenges. We elected not to change the nature of the study to focus on the impact of COVID-19 given the inclusion of prepandemic cohorts in the analysis and the commonality of stressors reported among all cohorts. Programs should be judicious regarding workload expectations and ensure a commensurate decrease in other responsibilities to safeguard the sustainability of participation. Flexibility in the face of unexpected shifts in participants’ personal or professional demands may also be advisable.

Some characteristics of the program, including its breadth, intensity, timing, and interdisciplinary nature may have also contributed to increased stress and burnout (Theme 4). Other programs may have differing program characteristics, requirements, structure, or support which may shift outcomes. A review of the published literature suggests that typical JFDPs are discipline-specific and of short duration (several days to a week in length).^21–24^ While we believe the multidisciplinary and year-long weekly structure of our program has benefits, its breadth and intensity may overwhelm participants while making comparisons to other programs challenging.

Amid evidence of worsening burnout and QoL during the program, some qualitative responses revealed that participants appreciated the program. All interviewees stated they would recommend the program to colleagues. While this may represent selection bias, reported benefits of skill building, networking, collaboration, and institutional knowledge were clearly seen in the data, despite the concomitant challenges. Our findings echo prior research demonstrating the protective effects of engagement^25^ and social connectedness^26^ against burnout and reinforce the potential for professional development programs to enhance key elements which prevent and mitigate burnout.

The majority of participants were women, a trend which reflects the composition of the cohorts that made up this study (2018-2019; 16 women, 14 men, 2019-2020; 22 women, 8 men, 2020-2021; 20 women, 12 men). Historically, participants in JFDP are split approximately 50% male and 50% female, but this breakdown varies from year to year, with a recent trend toward more female participants (approximately 55%-75% female). Burnout and WHC have both been shown to be more common among women.^4,27,28^ Because our analysis was descriptive, we cannot comment on whether female predominance in both pre- and post-surveys resulted in a meaningful influence on the finding of worsening burnout during the program.

Mentorship was highlighted as both beneficial (Theme 2) and problematic (Theme 4) by participants. This reinforces the importance of mentorship relationships in faculty development. Effective mentorship facilitated perspective taking and effective project navigation, while failure to connect with a mentor was cited as grounds for project abandonment and dissatisfaction. This suggests there may be value in additional training for both mentors and mentees to optimize this critical relationship in faculty development endeavors.

Several changes in the program have been made since the conclusion of this study in an attempt to reduce the stress of participation, starting with the 2023 to 2024 cohort. First, class start time was moved from 7 am to 8 am to assuage child care and other early start time challenges. Second, we increased the time to complete projects from one year to up to 2 years to allow participants to work on their own timeline. If extending their project for a second year, program leadership and individual mentors continue to provide support (through scheduled meetings, offered monthly). These additional meetings provide an ongoing opportunity for continued networking, peer-mentorship, and facilitated problem-solving. Participants continue to receive departmental support, although at a reduced FTE (0.05 FTE for Year 2.). Third, for participants who occasionally require remote participation due to travel, family obligations, or unexpected clinical obligations, we attempt to incorporate a remote option for all sessions, and when possible offer opportunities for make-up sessions so we can ensure that the content is covered. Fourth, to bolster the mentor–mentee relationship, we now offer mentor training at the beginning of the year, which is reviewed and revised each year based on feedback. Finally, we regularly check in with our participants regarding their mentors, and we have a formal mid-year check in with both mentors and mentees through surveys and meetings.

Limitations include a single site, small sample size, and lack of demographic data for qualitative interview participants. COVID-related changes influenced participants’ experiences and may limit generalizability of the results.

## Conclusions

JFDP participants demonstrated worsening of burnout and QoL during participation in the program, while benefiting from opportunities related to skill building and networking. The impact of such programs on the well-being of participants should be considered as an element of their design, evaluation, and refinement over time.
